# 

**Published:** 1874-07

**Authors:** 


### CONTENTS.

PAGE.

**CONTRIBUTIONS.**

Our Summer Resorts. BEN H. PRATT, A. M. ........... 247
Character and Long Life. P. H. HAYES, M. D. ..... 250
Digestion, Exercise & Sleep. W. C. COLEMAN, M.D. 253

**MEDICAL ITEMS AND NEWS.**

Night Sweats of Phthisis—Depilatory Mixture—Chilblains and Coryza—Syrp. Morphia Comp.—Neuralgia—Sugar and Magnesia an Antidote to Arsenic—Incontinence of Urine—Etherial Tincture of Phosphorus—Treatment of Cholera—Tasteless Cod Liver Oil—Flatus—Mouth Wash—Gonorrhœa—Ulcers—Burns—Hoarseness—Cholera Mixture—Ascarides—Expectorant Mixture—Liniment for Rheumatism—Tapeworm—Diabetes—Gonorrhœa, Gleet, &c.—Toothache—Prolapsus Ani—Abortive Treatment of Coryza—Angina Pectoris—Gastralgia—Opium Cure—Climate in Consumption—Hypodermic Injection of Carbolic Acid in Erysipelas—Spinal Anæmia—Treatment of Pulmonary Cavities through Chestwall—Vomiting of Pregnancy—Vomiting after meals.......256 to 262

**OUR CORRESPONDENCE.**

Eight Letters with Replies........ 262

PAGE.

**HOUSEHOLD HINTS AND RECIPES.**

To keep off Flies—Dandruff—Limpid and Flexible Varnish—Colic in a Filly—To keep away Mice—Cure for Balky Horse—Lotion for Fetid Feet—Copying Ink—Preserving Fence Posts—Mocking Bird Food—Cure for Scratches—To remove Garlic from Milk—Iced Apples—Wasp Stings—Horses Overheated—Distemper Cure—Killing Lice on Cattle—How to tell Counterfeits—Worcestershire Sauce—Baldness—Kalsomining Fluid—Milk Diet in Dysentery—Ether Glue—Cleaning Bottles—Roup Remedy—Cure for Stammering—Fly Paper—Bottled Ginger Beer ........ 264 to 266

**EDITORIAL.**

How we spent our Vacation ........ 267
Morbus Coxarius, or Hip Joint Disease........ 268
Muscæ Volitantes ........ 269
Catarrh ........ 270
Hot Weather........ 271

**CHIT-CHAT.**

A Beautiful Roe—All Aboard—Give it Up—Cataract—The *Gazette*—Why is It?—Cremation—Queer—Note It—New Medical Society—Academy Prize—Our Medical Items and News—Found at Last—Our Schools—That Thunder Storm—Surgical Novelties—The Hoboken Turtle Club—The Boys—Would'nt Set........271 to 273

